# 1-(2,6-Diiso­propyl­phen­yl)-1*H*-benzimidazole

**DOI:** 10.1107/S1600536813020473

**Published:** 2013-07-27

**Authors:** Sha Lai, Yong Chen, Yan Li, Yonggang Shen, Hongwei Wu

**Affiliations:** a Pharmaceutical Department of The First Affiliated Hospital of Guangdong Pharmaceutical University, Clinical Pharmacy Department of Guangdong Pharmaceutical University, Guangzhou 510080, People’s Republic of China

## Abstract

In the title compound, C_19_H_22_N_2_, both the benzimidazole unit and the 2,6-diiso­propyl­phenyl group are essentially planar [maximum deviations from the least-squares planes of 0.005 (1) and 0.009 (1) Å, respectively]. The dihedral angle between the two planes is 79.6 (7)°. In the crystal, mol­ecules are linked into chains along the *a*-axis direction by weak C—H⋯N inter­actions. The crystal structure also features C—H⋯π inter­actions, which link the chains into a three-dimensional network.

## Related literature

For the properties of related compounds, see: Shi *et al.* (2013 ▶); Cross *et al.* (1995 ▶); Akpinar *et al.* (2010 ▶); Wang *et al.* (2007 ▶); Mason *et al.* (1999 ▶). For bond lengths and angles in related structures, see: Jayamoorthy *et al.* (2013 ▶); Fathima *et al.* (2013 ▶); Geiger & Nellist (2013 ▶).
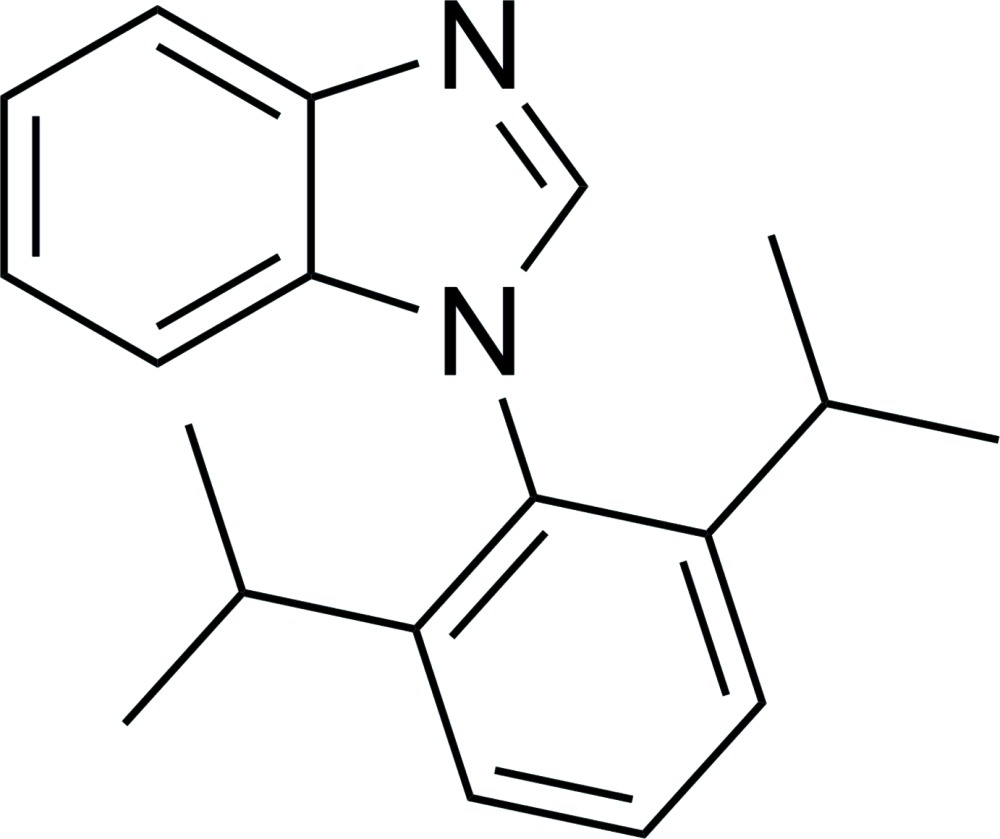


## Experimental

### 

#### Crystal data

C_19_H_22_N_2_
*M* *_r_* = 278.39
Orthorhombic, 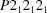

*a* = 6.6471 (8) Å
*b* = 14.1216 (18) Å
*c* = 17.285 (2) Å
*V* = 1622.5 (4) Å^3^
*Z* = 4
Mo *K*α radiation
μ = 0.07 mm^−1^
*T* = 173 K
0.44 × 0.42 × 0.28 mm

#### Data collection

Bruker APEXII CCD area-detector diffractometer
Absorption correction: multi-scan (*SADABS*; Bruker, 2008 ▶) *T*_min_ = 0.971, *T*_max_ = 0.982
9716 measured reflections
3551 independent reflections
3133 reflections with *I* > 2σ(*I*)
*R*_int_ = 0.020

#### Refinement

*R*[*F* ^2^ > 2σ(*F* ^2^)] = 0.035
*wR*(*F* ^2^) = 0.088
*S* = 1.08
3551 reflections
194 parameters
H-atom parameters constrained
Δρ_max_ = 0.16 e Å^−3^
Δρ_min_ = −0.16 e Å^−3^

### 

Data collection: *APEX2* (Bruker, 2008 ▶); cell refinement: *SAINT* (Bruker, 2008 ▶); data reduction: *SAINT*; program(s) used to solve structure: *SHELXS97* (Sheldrick, 2008 ▶); program(s) used to refine structure: *SHELXL97* (Sheldrick, 2008 ▶); molecular graphics: *SHELXTL* (Sheldrick, 2008 ▶); software used to prepare material for publication: *SHELXL97*.

## Supplementary Material

Crystal structure: contains datablock(s) I, New_Global_Publ_Block. DOI: 10.1107/S1600536813020473/bg2510sup1.cif

Structure factors: contains datablock(s) I. DOI: 10.1107/S1600536813020473/bg2510Isup2.hkl

Supplementary material file. DOI: 10.1107/S1600536813020473/bg2510Isup3.cml

Additional supplementary materials:  crystallographic information; 3D view; checkCIF report

## Figures and Tables

**Table 1:** Hydrogen-bond geometry (Å, °) *Cg*1 and *Cg*2 are the centroids of the C2–C7 and C8/C9/C13–C16 rings, respectively.

*D*—H⋯*A*	*D*—H	H⋯*A*	*D*⋯*A*	*D*—H⋯*A*
C6—H6⋯N1^i^	0.95	2.47	3.4040 (18)	168
C14—H14⋯*Cg*3^ii^	0.95	2.68	3.5908 (16)	150
C18—H18*B*⋯*Cg*2^iii^	0.98	2.79	3.5314 (17)	125

Symmetry codes: (i) 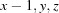; (ii) 
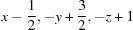
; (iii) 
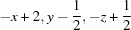
.

**Fig. 1.**
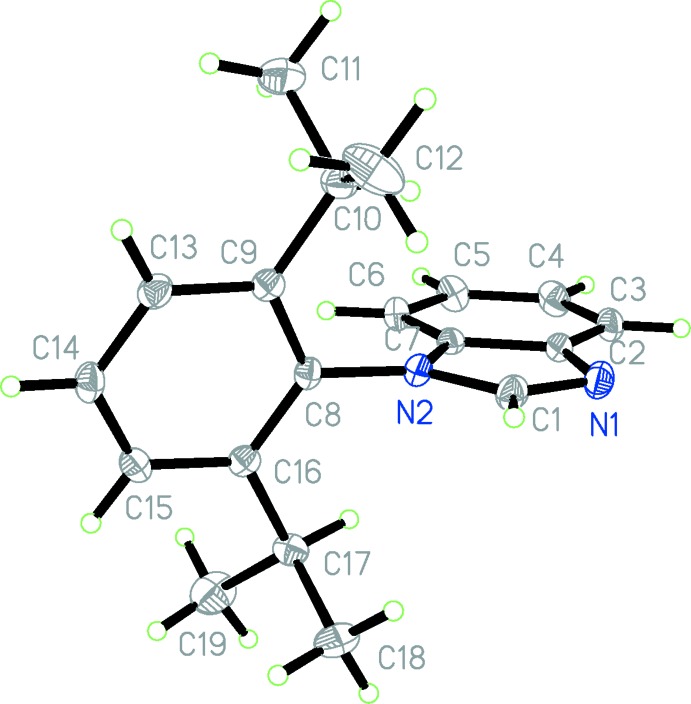
Molecular structure of the title compound. Displacement ellipsoids are shown at the 40% probability level. H atoms are presented as small spheres of arbitrary radius.

**Fig. 2.**
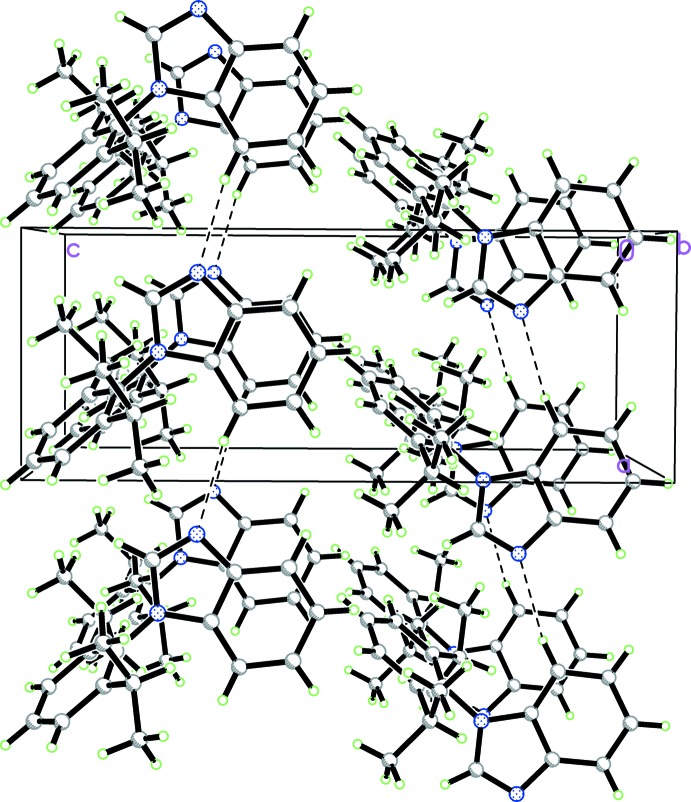
Packing diagram of (I). Dashed lines indicate intermolecular hydrogen bonding interactions.

## supplementary crystallographic information

### Comment

Benzimidazole is one of the most important organic intermediates in drug design, in light of the affinity it displays towards some enzymes and protein receptors (Mason *et al.*, 1999). Our interest is focused on the design and synthesis of benzimidazole derivatives with various ancillary ligands, and their application in antioxidant activities. Herein, we report the synthesis and structure of the title compound (I). Its molecular structure is shown in Fig.1. Bond lengths and angles of the benzimidazole group are in good agreement with those observed in other benzimidazole derivatives (Jayamoorthy *et al.*, 2013; Fathima *et al.*, 2013; Geiger *et al.*, 2013). Both the benzimidazole unit and the 2, 6-diisopropylphenyl groups are essentially planar (max. deviations from the L.S. plane: 0.005 (1) and 0.009 (1)Å, for atoms C7 and C9, respectively). The dihedral angle between both planes is 100.4° (7). In the crystal structure, the molecules are linked into chains along the *a* axis by intermolecular C—H···N hydrogen bonds (Table 1). The structure is further stabilized by weak intermolecular C—H···Cg interactions linking chains into a 3D network (Table 1 and Fig 2).

### Experimental

*N*-(2-bromophenyl)-N'-(2, 6-diisopropylphenyl)-Methanimidamide, (1.65 g, 4.58 mmol, 1 eq.) was dissolved in 18.5 ml DMSO. CuI (174 mg, 0.92 mmol, 20 mol%) and DBU (1.37 ml, 1.39 g, 9.16 mmol, 2 eq.) were added and the reaction was stirred for 3 h at 110 °C. H2O(160 ml) and acetoacetate (160 ml) were added and the layers were separated. The aqueous layer was extracted with acetoacetate (2 τimes 20 ml), the combined organic layers were dried over Na2SO4. The crude mixture was purified by column chromatography to afford the benzimidazole (1.22 g, 96%). Single crystals were grown in ethanol as a solvent at room temperature.

### Refinement

C-bound H-atoms were geometrically positioned (C–H 0.93 or 0.98 Å for aromatic or methyl C atoms respectively) and refined using a riding model, with *U*_iso_ = 1.2/1.5*U*_eq_ (C), respectively.

### Crystal data

**Table:**

C_19_H_22_N_2_	*F*(000) = 600
*M**_r_* = 278.39	*D*_x_ = 1.140 Mg m^−^^3^
Orthorhombic, *P*2_1_2_1_2_1_	Mo *K*α radiation, λ = 0.71073 Å
Hall symbol: P 2ac 2ab	Cell parameters from 5376 reflections
*a* = 6.6471 (8) Å	θ = 2.4–27.1°
*b* = 14.1216 (18) Å	µ = 0.07 mm^−^^1^
*c* = 17.285 (2) Å	*T* = 173 K
*V* = 1622.5 (4) Å^3^	Block, colorless
*Z* = 4	0.44 × 0.42 × 0.28 mm

### Data collection

**Table:**

Bruker APEXII CCD area-detector diffractometer	3551 independent reflections
Radiation source: fine-focus sealed tube	3133 reflections with *I* > 2σ(*I*)
Graphite monochromator	*R*_int_ = 0.020
Detector resolution: 0 pixels mm^-1^	θ_max_ = 27.1°, θ_min_ = 1.9°
phi and ω scans	*h* = −8→8
Absorption correction: multi-scan (*SADABS*; Bruker, 2008)	*k* = −18→16
*T*_min_ = 0.971, *T*_max_ = 0.982	*l* = −18→22
9716 measured reflections	

### Refinement

**Table:**

Refinement on *F*^2^	Primary atom site location: structure-invariant direct methods
Least-squares matrix: full	Secondary atom site location: difference Fourier map
*R*[*F*^2^ > 2σ(*F*^2^)] = 0.035	Hydrogen site location: inferred from neighbouring sites
*wR*(*F*^2^) = 0.088	H-atom parameters constrained
*S* = 1.08	*w* = 1/[σ^2^(*F*_o_^2^) + (0.0411*P*)^2^ + 0.2264*P*] where *P* = (*F*_o_^2^ + 2*F*_c_^2^)/3
3551 reflections	(Δ/σ)_max_ < 0.001
194 parameters	Δρ_max_ = 0.16 e Å^−^^3^
0 restraints	Δρ_min_ = −0.16 e Å^−^^3^

### Special details

**Table:**

Geometry. All e.s.d.'s (except the e.s.d. in the dihedral angle between two l.s. planes) are estimated using the full covariance matrix. The cell e.s.d.'s are taken into account individually in the estimation of e.s.d.'s in distances, angles and torsion angles; correlations between e.s.d.'s in cell parameters are only used when they are defined by crystal symmetry. An approximate (isotropic) treatment of cell e.s.d.'s is used for estimating e.s.d.'s involving l.s. planes.

### Fractional atomic coordinates and isotropic or equivalent isotropic displacement parameters (Å^2^)

**Table:**

	*x*	*y*	*z*	*U*_iso_*/*U*_eq_	
C1	1.2274 (2)	0.79599 (10)	0.29080 (8)	0.0303 (3)	
H1	1.2920	0.7885	0.3394	0.036*	
N1	1.32623 (17)	0.80167 (9)	0.22581 (7)	0.0335 (3)	
C2	1.17791 (19)	0.81252 (9)	0.17010 (8)	0.0262 (3)	
N2	1.02227 (16)	0.80177 (8)	0.28307 (6)	0.0243 (2)	
C3	1.1940 (2)	0.82181 (10)	0.08970 (8)	0.0327 (3)	
H3	1.3217	0.8218	0.0650	0.039*	
C4	1.0203 (2)	0.83093 (11)	0.04764 (8)	0.0366 (3)	
H4	1.0285	0.8375	−0.0070	0.044*	
C5	0.8308 (2)	0.83080 (11)	0.08327 (8)	0.0347 (3)	
H5	0.7138	0.8373	0.0522	0.042*	
C6	0.8096 (2)	0.82147 (10)	0.16281 (8)	0.0284 (3)	
H6	0.6814	0.8211	0.1872	0.034*	
C7	0.9866 (2)	0.81270 (8)	0.20455 (7)	0.0234 (3)	
C8	0.87636 (19)	0.80084 (9)	0.34461 (7)	0.0227 (3)	
C9	0.8510 (2)	0.88320 (9)	0.38878 (7)	0.0254 (3)	
C10	0.9634 (2)	0.97431 (10)	0.37135 (8)	0.0324 (3)	
H10	1.0380	0.9652	0.3217	0.039*	
C11	0.8185 (3)	1.05673 (13)	0.36039 (13)	0.0604 (6)	
H11A	0.7243	1.0419	0.3185	0.091*	
H11B	0.8946	1.1140	0.3472	0.091*	
H11C	0.7436	1.0674	0.4084	0.091*	
C12	1.1172 (4)	0.99708 (15)	0.43400 (12)	0.0645 (6)	
H12A	1.0480	1.0063	0.4835	0.097*	
H12B	1.1898	1.0550	0.4202	0.097*	
H12C	1.2127	0.9445	0.4386	0.097*	
C13	0.7136 (2)	0.87915 (10)	0.44978 (8)	0.0303 (3)	
H13	0.6941	0.9334	0.4815	0.036*	
C14	0.6056 (2)	0.79775 (11)	0.46485 (8)	0.0315 (3)	
H14	0.5136	0.7963	0.5069	0.038*	
C15	0.6306 (2)	0.71811 (10)	0.41892 (8)	0.0292 (3)	
H15	0.5532	0.6630	0.4292	0.035*	
C16	0.76747 (19)	0.71788 (10)	0.35795 (7)	0.0249 (3)	
C17	0.8059 (2)	0.62843 (10)	0.31125 (8)	0.0303 (3)	
H17	0.8567	0.6479	0.2592	0.036*	
C18	0.9704 (3)	0.56985 (11)	0.35044 (10)	0.0421 (4)	
H18A	1.0924	0.6083	0.3557	0.063*	
H18B	0.9999	0.5138	0.3189	0.063*	
H18C	0.9244	0.5499	0.4018	0.063*	
C19	0.6166 (3)	0.56884 (13)	0.29914 (12)	0.0534 (5)	
H19A	0.5719	0.5432	0.3489	0.080*	
H19B	0.6465	0.5166	0.2636	0.080*	
H19C	0.5101	0.6085	0.2771	0.080*	

### Atomic displacement parameters (Å^2^)

**Table:**

	*U*^11^	*U*^22^	*U*^33^	*U*^12^	*U*^13^	*U*^23^
C1	0.0239 (6)	0.0333 (8)	0.0338 (7)	0.0025 (6)	−0.0026 (6)	0.0005 (6)
N1	0.0241 (6)	0.0388 (7)	0.0375 (7)	0.0032 (5)	0.0041 (5)	0.0009 (5)
C2	0.0238 (6)	0.0226 (6)	0.0324 (7)	−0.0001 (5)	0.0043 (5)	−0.0012 (5)
N2	0.0211 (5)	0.0284 (6)	0.0234 (5)	0.0027 (5)	0.0009 (4)	−0.0005 (4)
C3	0.0331 (7)	0.0323 (8)	0.0325 (7)	−0.0025 (6)	0.0124 (6)	−0.0023 (6)
C4	0.0451 (9)	0.0407 (8)	0.0239 (7)	−0.0053 (7)	0.0070 (7)	−0.0005 (6)
C5	0.0328 (8)	0.0425 (8)	0.0286 (7)	−0.0065 (7)	−0.0041 (6)	0.0019 (6)
C6	0.0232 (6)	0.0348 (8)	0.0271 (7)	−0.0021 (6)	0.0015 (5)	−0.0005 (6)
C7	0.0265 (6)	0.0193 (6)	0.0244 (6)	−0.0011 (5)	0.0041 (5)	−0.0013 (5)
C8	0.0197 (6)	0.0294 (7)	0.0189 (6)	0.0034 (5)	−0.0001 (5)	0.0015 (5)
C9	0.0260 (6)	0.0271 (7)	0.0232 (6)	0.0022 (5)	−0.0026 (5)	0.0014 (5)
C10	0.0404 (8)	0.0262 (7)	0.0305 (7)	−0.0017 (6)	0.0044 (6)	−0.0009 (5)
C11	0.0707 (13)	0.0352 (9)	0.0754 (13)	0.0121 (9)	0.0173 (11)	0.0173 (9)
C12	0.0756 (14)	0.0544 (12)	0.0634 (13)	−0.0329 (11)	−0.0177 (11)	0.0042 (9)
C13	0.0354 (7)	0.0303 (7)	0.0254 (7)	0.0052 (6)	0.0029 (6)	−0.0026 (6)
C14	0.0283 (7)	0.0431 (8)	0.0232 (7)	0.0030 (6)	0.0054 (5)	0.0025 (6)
C15	0.0271 (7)	0.0322 (7)	0.0283 (7)	−0.0034 (6)	−0.0003 (6)	0.0056 (6)
C16	0.0249 (6)	0.0274 (7)	0.0226 (6)	0.0028 (5)	−0.0039 (5)	0.0014 (5)
C17	0.0374 (8)	0.0274 (7)	0.0262 (7)	−0.0015 (6)	−0.0019 (6)	−0.0018 (5)
C18	0.0470 (10)	0.0309 (8)	0.0485 (9)	0.0066 (7)	−0.0081 (8)	−0.0062 (7)
C19	0.0466 (10)	0.0429 (10)	0.0706 (13)	−0.0067 (8)	−0.0106 (10)	−0.0192 (9)

### Geometric parameters (Å, º)

**Table:**

C1—N1	1.3038 (18)	C11—H11A	0.9800
C1—N2	1.3725 (16)	C11—H11B	0.9800
C1—H1	0.9500	C11—H11C	0.9800
N1—C2	1.3868 (18)	C12—H12A	0.9800
C2—C3	1.400 (2)	C12—H12B	0.9800
C2—C7	1.4044 (18)	C12—H12C	0.9800
N2—C7	1.3865 (16)	C13—C14	1.380 (2)
N2—C8	1.4396 (15)	C13—H13	0.9500
C3—C4	1.370 (2)	C14—C15	1.387 (2)
C3—H3	0.9500	C14—H14	0.9500
C4—C5	1.402 (2)	C15—C16	1.3924 (19)
C4—H4	0.9500	C15—H15	0.9500
C5—C6	1.388 (2)	C16—C17	1.5207 (19)
C5—H5	0.9500	C17—C19	1.528 (2)
C6—C7	1.3857 (18)	C17—C18	1.529 (2)
C6—H6	0.9500	C17—H17	1.0000
C8—C16	1.3963 (19)	C18—H18A	0.9800
C8—C9	1.4013 (18)	C18—H18B	0.9800
C9—C13	1.3961 (19)	C18—H18C	0.9800
C9—C10	1.5181 (19)	C19—H19A	0.9800
C10—C11	1.522 (2)	C19—H19B	0.9800
C10—C12	1.524 (2)	C19—H19C	0.9800
C10—H10	1.0000		
			
N1—C1—N2	114.39 (12)	C10—C11—H11C	109.5
N1—C1—H1	122.8	H11A—C11—H11C	109.5
N2—C1—H1	122.8	H11B—C11—H11C	109.5
C1—N1—C2	104.30 (11)	C10—C12—H12A	109.5
N1—C2—C3	130.20 (12)	C10—C12—H12B	109.5
N1—C2—C7	110.46 (11)	H12A—C12—H12B	109.5
C3—C2—C7	119.33 (12)	C10—C12—H12C	109.5
C1—N2—C7	105.78 (11)	H12A—C12—H12C	109.5
C1—N2—C8	126.64 (11)	H12B—C12—H12C	109.5
C7—N2—C8	127.52 (11)	C14—C13—C9	121.13 (13)
C4—C3—C2	118.11 (13)	C14—C13—H13	119.4
C4—C3—H3	120.9	C9—C13—H13	119.4
C2—C3—H3	120.9	C13—C14—C15	120.34 (12)
C3—C4—C5	121.57 (13)	C13—C14—H14	119.8
C3—C4—H4	119.2	C15—C14—H14	119.8
C5—C4—H4	119.2	C14—C15—C16	120.90 (13)
C6—C5—C4	121.77 (14)	C14—C15—H15	119.5
C6—C5—H5	119.1	C16—C15—H15	119.5
C4—C5—H5	119.1	C15—C16—C8	117.50 (12)
C7—C6—C5	115.96 (12)	C15—C16—C17	120.90 (12)
C7—C6—H6	122.0	C8—C16—C17	121.48 (11)
C5—C6—H6	122.0	C16—C17—C19	113.06 (12)
C6—C7—N2	131.68 (12)	C16—C17—C18	109.53 (11)
C6—C7—C2	123.26 (11)	C19—C17—C18	110.57 (13)
N2—C7—C2	105.06 (11)	C16—C17—H17	107.8
C16—C8—C9	122.96 (11)	C19—C17—H17	107.8
C16—C8—N2	118.61 (11)	C18—C17—H17	107.8
C9—C8—N2	118.43 (11)	C17—C18—H18A	109.5
C13—C9—C8	117.14 (12)	C17—C18—H18B	109.5
C13—C9—C10	120.43 (12)	H18A—C18—H18B	109.5
C8—C9—C10	122.42 (12)	C17—C18—H18C	109.5
C9—C10—C11	111.19 (13)	H18A—C18—H18C	109.5
C9—C10—C12	111.60 (13)	H18B—C18—H18C	109.5
C11—C10—C12	110.58 (15)	C17—C19—H19A	109.5
C9—C10—H10	107.8	C17—C19—H19B	109.5
C11—C10—H10	107.8	H19A—C19—H19B	109.5
C12—C10—H10	107.8	C17—C19—H19C	109.5
C10—C11—H11A	109.5	H19A—C19—H19C	109.5
C10—C11—H11B	109.5	H19B—C19—H19C	109.5
H11A—C11—H11B	109.5		
			
N2—C1—N1—C2	0.30 (16)	C7—N2—C8—C9	−100.77 (14)
C1—N1—C2—C3	−179.65 (15)	C16—C8—C9—C13	1.78 (19)
C1—N1—C2—C7	−0.23 (15)	N2—C8—C9—C13	−177.79 (12)
N1—C1—N2—C7	−0.25 (16)	C16—C8—C9—C10	−177.13 (12)
N1—C1—N2—C8	−177.75 (12)	N2—C8—C9—C10	3.30 (18)
N1—C2—C3—C4	179.48 (14)	C13—C9—C10—C11	−54.09 (19)
C7—C2—C3—C4	0.1 (2)	C8—C9—C10—C11	124.78 (15)
C2—C3—C4—C5	−0.2 (2)	C13—C9—C10—C12	69.89 (19)
C3—C4—C5—C6	0.0 (2)	C8—C9—C10—C12	−111.24 (16)
C4—C5—C6—C7	0.2 (2)	C8—C9—C13—C14	−1.0 (2)
C5—C6—C7—N2	−179.57 (13)	C10—C9—C13—C14	177.94 (13)
C5—C6—C7—C2	−0.32 (19)	C9—C13—C14—C15	−0.5 (2)
C1—N2—C7—C6	179.44 (14)	C13—C14—C15—C16	1.4 (2)
C8—N2—C7—C6	−3.1 (2)	C14—C15—C16—C8	−0.60 (19)
C1—N2—C7—C2	0.09 (14)	C14—C15—C16—C17	175.54 (13)
C8—N2—C7—C2	177.56 (12)	C9—C8—C16—C15	−1.00 (18)
N1—C2—C7—C6	−179.34 (13)	N2—C8—C16—C15	178.57 (11)
C3—C2—C7—C6	0.16 (19)	C9—C8—C16—C17	−177.12 (12)
N1—C2—C7—N2	0.09 (14)	N2—C8—C16—C17	2.45 (18)
C3—C2—C7—N2	179.58 (12)	C15—C16—C17—C19	36.52 (19)
C1—N2—C8—C16	−103.40 (15)	C8—C16—C17—C19	−147.49 (14)
C7—N2—C8—C16	79.63 (16)	C15—C16—C17—C18	−87.28 (16)
C1—N2—C8—C9	76.19 (17)	C8—C16—C17—C18	88.71 (15)

### Hydrogen-bond geometry (Å, º)

Cg1 and Cg2 are the centroids of the C2–C7 and C8/C9/C13–C16 rings, respectively.

**Table:**

*D*—H···*A*	*D*—H	H···*A*	*D*···*A*	*D*—H···*A*
C6—H6···N1^i^	0.95	2.47	3.4040 (18)	168
C14—H14···*Cg*3^ii^	0.95	2.68	3.5908 (16)	150
C18—H18*B*···*Cg*2^iii^	0.98	2.79	3.5314 (17)	125

Symmetry codes: (i) *x*−1, *y*, *z*; (ii) *x*−1/2, −*y*+3/2, −*z*+1; (iii) −*x*+2, *y*−1/2, −*z*+1/2.
